# So Far, So Close: Identification with Proximal and Distal Groups as a Resource in Dealing with the COVID-19 Pandemic

**DOI:** 10.3390/ijerph191811231

**Published:** 2022-09-07

**Authors:** Anna Rita Graziani, Lucia Botindari, Michela Menegatti, Silvia Moscatelli

**Affiliations:** 1 Department of Communication and Economics, University of Modena and Reggio Emilia, Viale Allegri 9, 42121 Reggio Emilia, Italy; 2 SAIS Europe, Johns Hopkins University, Via Andreatta 3, 40126 Bologna, Italy; 3 Department of Psychology, University of Bologna, Viale Berti Pichat 5, 40127 Bologna, Italy

**Keywords:** social identification, proximal groups, distal groups, COVID-19, pandemic, compliance with rules, prosocial behaviors, emotions, vaccination

## Abstract

A robust body of research has highlighted the fundamental role of social identifications in dealing with emergencies and in predicting commitment behaviors. We report the results of two studies carried out in Italy to assess whether the subjective sense of belonging to meaningful proximal and distal social groups affected people’s ability to cope with the pandemic crisis. Study 1 (*N* = 846) shows that different identifications with proximal (i.e., family and friends) and distal social groups (i.e., nation, European, and humankind) may act as buffers for individuals by reducing negative emotions and negative expectations about the future after COVID-19 and by increasing people’s intentions to adhere to containment measures and to be involved in prosocial actions. Study 2 (*N* = 350) highlights the role of European identification in predicting propensities for using the tracing app and getting vaccinated. These results confirm the benefits of various types of identification (proximal vs. distant) in helping individuals deal with the COVID-19 pandemic.

## 1. Introduction

Since the end of 2019, when the first cases of COVID-19 were identified in Wuhan, China, the SARS-CoV-2 spread rapidly, ravaging most countries of the world. A few months later, on 11 March 2020, the World Health Organization (WHO) declared pandemic status. In Italy, where the first case of evident SARS-CoV-2 transmission was diagnosed on 20 February, the virus spread quickly and, at the beginning of March 2020, Italy exhibited the world’s highest rate of coronavirus infection [1]. In response to the growing pandemic, the Italian government imposed a strict nationwide lockdown on 9 March 2020: Activities of schools and universities, social events, and gatherings (e.g., team sports, religious celebrations, cinemas, museums, etc.) were suspended; all non-essential industries and businesses were halted; members of the public were confined to their homes, except in instances of emergencies, essential work, or health issues. A 6 pm curfew was imposed, and smart working was encouraged. People were asked to wear face masks in public places, keep physical distance, and rub their hands with alcohol-based sanitizers and disinfectants. These social limitations were loosened on 16 May 2020.

Despite being widely approved by public opinion [2], these urgent measures imposed substantial limitations on individual freedom and required behaviors that go against shared social norms, such as maintaining physical distance with ingroup members, especially in the case of family and friends [3]. However, the success of the fight against a pandemic greatly depends on citizens’ adherence to such restrictive policies and their intentions to engage in protective behaviors, such as getting vaccinated and using tracing apps designed to indicate possible risky contacts. For this reason, it is important to shed light on the factors that can increase individuals’ ability to cope with the pandemic and their compliance with containment measures.

One possible factor that can predict engagement in protecting behaviors and the avoidance of potentially harmful behaviors is social identity [4]. Studies in the field have confirmed the crucial role of social identifications in helping human beings make sense of the world and face threats by means of enhancing people’s senses of belonging and support, meaning and purpose, control and agency, and collective self-esteem (e.g., [5]).

We present two studies conducted in Italy, which apply the social identity framework [6] in examining whether the subjective sense of belonging to various meaningful social groups (family, friends, nation, Europe, and humankind) positively influenced people’s coping strategies against COVID-19. The studies were carried out in different stages of the pandemic evolution, characterized by different knowledge and strategies to fight the coronavirus infection. Study 1 was conducted during the Italian lockdown and explored the role of different identifications in dealing with negative emotions, encouraging protective and altruistic behaviors, and diminishing negative expectations about the future. Study 2 was conducted when the containment measures were partially loosened, and the public debate was concentrated on the ongoing development of a tracing app and vaccination against COVID-19; therefore, it focused on the role of social identifications in affecting the intention to adopt these new measures to contain the spread of the pandemic.

## 2. Literature Review and Research Overview

### 2.1. Dealing with Coronavirus through Social Identification

According to the social identity tradition [6], belonging to a social group constitutes a fundamental resource for human beings. Group membership fulfills individuals’ needs for control and agency, a sense of belonging and social support, self-esteem, uncertainty reduction, and meaning [7,8,9,10,11]. Moreover, being part of a group has a positive impact on individuals’ health and well-being [12,13,14], protects against and alleviates depression symptoms [15], reduces post-traumatic stress [16], and facilitates the transitions in important life changes [17]. The role of social identification in reducing stress and promoting life satisfaction emerged in different contexts, for instance, when individuals face severe illnesses or work strain [18].

These positive effects are bolstered for individuals who belong to multiple groups [19,20]. The greater the number of group memberships individuals have access to, the more social and psychological resources they can recruit from their connections to similar others [21,22]. Research has consistently shown that having multiple identifications further promotes psychological well-being and social engagement [23,24], mental and physical resilience [25], and improves quality of life through coping strategies [21] and social adaptation [17]. Both proximal groups (such as family and close friends) and distal groups are relevant sources of psychological well-being and adjustment [26]. This reasoning is corroborated from a sociological point of view, according to which groups with strong ties (e.g., family, close friends) are essential for one’s life, while belonging to groups characterized by more tenuous connections can be even more important in carrying new information and perspective [27]. The positive effects of the “social cure” provided by group memberships emerged in coping with the uncertainty and threat induced by COVID-19 [4,28]. A study conducted in 67 countries reported that national identification was positively related to mental well-being as individuals dealt with the loneliness elicited by anti-contagion policies such as lockdowns and mass quarantines [29]. Other studies provided additional confirmation of the potential of having multiple identifications. Paolini et al. [30] found that national identification and identification with humankind positively influenced well-being and happiness during the lockdown in Italy. Similarly, Vignoles et al. [4] showed that community and humankind identification were predictive of lower incidence of depression symptoms during the UK’s early lockdown.

Moreover, when a social identity is salient, individuals consider themselves group members, which in turn increases their commitment toward the group and motivates them to accord with its norms [31,32]. This is particularly true when people deal with a common threat, such as the COVID-19 pandemic [28]. Vignoles et al. [4] showed that identifications with different social groups mobilize resilient responses against COVID-19: Family identification promotes the respect for physical distancing, while community and national identifications positively influence prosocial actions.

These senses of shared identities increase trust toward other group members [33,34]. In a study conducted in Italy, Moscatelli et al. [35] found that higher national and European identifications fueled respondents’ optimistic expectations about the post-pandemic future, leading to the improvement of governments and humankind. The effects of national and European identifications on the positive views of the future have been explained by reference to increased trust in political institutions, such as the Italian government and the EU. Interestingly, national, and European identifications also played important roles in buffering individuals’ wishes for strong leaders in response to the COVID-19 crisis [36]. National identification has been associated with a decreased wish for strong leadership, due to heightened trust in other people, health authorities, and the scientific community. Identifying oneself as European is likely to play a more direct role in buffering individuals’ wishes for strong leadership in response to the pandemic crisis [36]. The aim in both of our present studies is to examine whether identifications with different social groups helped individuals to deal with the COVID-19 pandemic.

### 2.2. Overview of the Current Studies

We present two studies investigating the role of multiple identities in predicting responses to the COVID-19 pandemic; our focus is on identifications with family, friends, Italy, Europe, and humankind. In choosing these specific reference groups, we consider two important aspects. Firstly, we take into account group differences in the level of social proximity or distance, that is, familiarity and emotional closeness with other ingroup members [37]. In this sense, family and friends can be conceived as proximal groups in which each member directly interacts with every other member. The broader groups (i.e., nation, Europe, and humankind) are more distal, because no member in any of these groups can directly connect and interact with every other member of that group [38]. Studies in the field have shown that a proximal group tends to exert a stronger normative influence on individuals than a distal group can [39,40]. However, given that the pandemic is a global phenomenon, and we are all in this together [41], it is possible that these distal identifications were perceived to be particularly relevant and so played an important role in predicting normative behaviors.

Secondly, we focus on groups that, as mentioned, were particularly touched by the pandemic and were involved in the containment policies [4]. Since the beginning of the COVID-19 outbreak, the closeness and the connection between lower-risk and higher-risk age groups made family and friends particularly vulnerable to the contagion [4]. Many of the containment measures adopted by governments limited the movement and the physical interactions between people, thereby going against certain group norms (e.g., regularly making efforts to be physically close to one’s relatives). However, maintaining physical distance from ingroup members such as friends and family is both a challenge and a threat, because being close to in-group members has many benefits, including making one feel safer [3].

National identity is important in this context because the containment measures were decided and promoted at a national level. Moreover, national identity is related to individual well-being [4,30], as well as support for, and engagement in, protecting behaviors around the word [42]. Several religious and political leaders, including Pope Francis [43] and New Zealand Prime Minister Jacinda Ardern [44], employed successful communication strategies characterized by an emphasis on national identity as leverage for obtaining greater compliance with the new restrictions while preserving optimism and hope for the future. At the end of the speech announcing the first general lockdown, Italian prime minister Giuseppe Conte said, “We are all part of the same community […]. We keep distant today to hug tighter tomorrow. We will run together again; we will make it together.” Vignoles et al. [4] have suggested that linking the protective actions and behaviors to the national identity was the key to mobilizing positive responses to COVID-19.

European and humankind identifications are important identities because the pandemic is a global phenomenon with no respect for borders. For this reason, many leaders have highlighted the importance of coordination, cooperation, and solidarity among countries, so as to reduce the spread of the virus and mitigate its long-term societal and economic consequences (e.g., [45]).

Following this line of thought, we conducted two studies during different stages of the pandemic spread to analyze the role of social identifications in buffering the negative consequences of COVID-19 and in promoting protective behaviors in line with the coronavirus’ evolution. Study 1 was conducted during the lockdown, when Italy was the worst-hit country after China and there were no proven treatments for COVID-19. In this situation of great uncertainty, people had to change their life dramatically to prevent the spread of the infection. Considering this peculiar situation, Study 1 focuses on the associations between proximal and distal identifications and emotional, behavioral, and attitudinal outcomes related to the pandemic. In particular, it investigates the role of social identification in reducing the negative emotions triggered by the COVID-19 crisis and encouraging protective and prosocial behaviors while contrasting negative expectations about the possible repercussions of the pandemic.

Study 2 was conducted when the containment measures were partially loosened, and people were slowly going back to normal life. At that time, social distancing and other protective behaviors were still recommended, but new measures for the contagion containment were being in development. The Italian government asked citizens to employ a tracing app to limit the spread of the virus. Moreover, since scientists were moving at record speed to create new vaccines, implementing an effective vaccination campaign was the next step to facing the virus. Thus, Study 2 examines the relationships between social identifications, emotions triggered by COVID-19, and the intention to use a contact-tracing app and to get vaccinated.

## 3. Study 1

One premise of social identity theory is that the subjective sense of belonging to a social group protects against adverse psychosocial effects of crises (e.g., [12,28]). Based on this, we examine the role of the different identifications in helping individuals to cope with the coronavirus pandemic. Firstly, we assess whether proximal (family and friends) and distal (nation, Europe, and humankind) identifications relate to reduced negative emotional reactions to COVID-19. Secondly, we seek to identify those patterns of social identifications that are associated with behaviors opposing the contagion, such as engaging in protecting behaviors (e.g., maintaining social distance) or seeking information about the pandemic. Since part of social life was maintained online due to the strict lockdown, we also focus on respondents’ engagement in online actions against the pandemic (e.g., writing or sharing posts encouraging compliance with the containment rules on social networking sites). Thirdly, we analyzed how different patterns of social identifications predicted prosocial behaviors toward people in need during the lockdown. Fourthly, we examine whether proximal and distal identifications were related to reductions in negative expectations regarding the post-COVID-19 future. Finally, we investigate the role of negative emotions as mediators of the effects of social identifications on these outcome variables.

## 4. Method

### 4.1. Participants

A total of 1146 Italian participants, recruited through social networking sites and snowball sampling, volunteered for the study, which was presented as research on the psychosocial correlates of the pandemic. Four participants did not provide consent, and 296 were excluded for failing to complete the entire questionnaire. The final sample consists of 846 participants (612 women, 234 men; *M*_age_ = 38.32 years; *SD* = 14.90 years; range 18–79 years). Of these, 524 live in Northern Italy, and 322 live in Central and Southern Italy. A total of 58 participants (6.9%) reported that they had contracted the coronavirus; 191 (22.6%) indicated that a family member or a close friend had contracted the virus. A sensitivity analysis conducted with G*Power showed that our sample was sufficient to detect small-size effects of *f*^2^ = 0.03 (corresponding to *R*^2^ = 0.03), assuming an alpha of 0.05, and power of 0.95.

### 4.2. Procedure

The project was approved by the Bioethical Committee of the University of Bologna. The questionnaire was administered via Qualtrics between 15 and 30 April 2020. After providing their consent to participate, respondents completed measures of identification with proximal (family and friends) and distal (one’s nation, Europe, and humankind) groups. They were then presented with measures of negative emotional reactions to the COVID-19 pandemic, protective behaviors (compliance with the containment rules, search for information, or engagement in online actions) and pro-social behaviors (offering material assistance or emotional support to people in need). Participants also reported measures of expectations about the pandemic’s negative repercussions in terms of their social lives, their economic circumstances, as well as the Italian and global economies. The questionnaire includes other measures that are not analyzed in the present paper (e.g., positive expectations about the future, coping strategies). Findings from the entire research project were already included in a previous publication [35], which focused on a different issue and tested different hypotheses. Two of the measures considered here (identification with Italians and identification with Europeans) were included in the publication.

Participants were asked whether they, a member of their family, or a close friend had contracted the coronavirus. Responses to the two questions are collapsed in a “personal experience with COVID-19” variable. Overall, 224 respondents (26.5%) reported having had a personal experience with COVID-19.

Participants were then asked to report their political orientation on an 11-point left–right scale (0 = extremely left, 10 = extremely right; *M* = 3.99, *SD* = 2.20), and provided demographic information, including their region of residence. Given that Italy’s northern regions were severely hit by the coronavirus at the time of data collection, whereas its central and southern regions had much fewer cases, we divide respondents’ places of residence into northern regions and central-southern regions. 

### 4.3. Measures

**Social identification.** We adapted Sani et al.’s [46] Group Identification Scale to measure identification with family or friends, as well as national and European identifications. Each measure consists of three items addressing the respondent’s sense of belonging to the group, e.g., “I have a sense of belonging to my family/my group of close friends/Italians/Europeans” (1 = strongly disagree; 7 = strongly agree). Cronbach’s alphas are α = 0.82 for identification with ones’ family, α = 0.88 for identification with friends, α = 0.82 for national identification, and α = 0.91 for European identification. Humankind identification was measured by adapting Albarello et al.’s [26] measure and consisted of three items, e.g., “I identify with all human beings” (α = 0.82).

**Negative Emotional Reactions.** Data on negative emotions were gathered by asking respondents the extent to which, thinking of the COVID-19 pandemic, they felt nervous, upset, worried, distressed, or fearful (1= not at all; 5 = very much). An index of negative emotional reactions is calculated as a mean of five items (α = 0.75).

**Protective and prosocial behaviors.** The measures concerning protective and prosocial behaviors in response to coronavirus were introduced by the question, “How often did or do you engage in the following behaviors?” (1 = never; 5 = very often). Two items measure compliance with the COVID-19 containment rules: “I respect the prescriptions imposed by the Ministry of Health to avoid contagion (e.g., Wash hands frequently; Maintain social distancing)” and, “I respect the prescriptions imposed by the Ministry of the Interior concerning movements (e.g., leaving the house for essential reasons only)” (α = 0.71).

Three items measured search for information: “I search for information on the symptoms of COVID-19 infection on the web,” “I read in-depth articles on the pandemic, either on paper magazines or on the web,” and, “I watch newscasts or other tv shows to learn more about COVID-19” (α = 0.71). Engagement in online actions against the pandemic was measured through two items concerning online sharing of recommendations relating to containment of the virus: “I have personally written or shared posts encouraging compliance with the containment rules on social networking sites,” and, “I have shared messages or videos made by healthcare staff or scientists to help people face with the pandemic” (α = 0.72).

Finally, two items measured the extent to which participants helped people in need: “I have helped older people or other needy people deal with their material needs (e.g., buying drugs, doing shopping),” and, “I try to offer moral support to people in trouble” (α = 0.53).

**Negative expectations regarding the future.** Expectations about the negative consequences of the pandemic were measured through five ad-hoc items, preceded by the following instruction: “Thinking of the possible repercussions of the COVID-19 emergency in the next months, please rate the extent to which you disagree or agree with the following items” (1 = completely disagree; 7 = completely agree). Three items were concerned with expectations about one’s social life: “The pandemic will have a negative influence on my social life,” “The COVID-19 will render people more wary in interacting with others,” and, “The pandemic will make social relationships harder” (α = 0.75). Two items referred to the economic consequences of the pandemic at the personal level: “The pandemic will negatively influence the economic status of my family,” and, “The pandemic will negatively impact on my work/my search for work” (α = 0.61). Two items concerned the pandemic’s impact on the Italian and global economies, e.g., “The pandemic will have a negative impact on the [Italian/global] economy” (α = 0.75).

## 5. Results

### 5.1. Preliminary Analyses

Table 1 reports descriptive statistics on the total sample as a function of gender, place of residence, and personal experience with COVID-19 infection. In general terms, it is interesting to note that identification with one’s family was higher than identification with all other groups (ts > 10.81, ps < 0.001, Cohen’s *d*s > 0.37). Identification with friends was higher than both national and European identifications (ts > 10.17, ps < 0.001, *d*s > 0.375), whereas it did not differ from humankind, t(845) = 0.86, p = 0.389. Humankind identification was higher than national and European identification (ts > 8.57, *p* < 0.001, *d*s > 0.30).

We conducted a series of *t*-tests to assess variations in the study measures due to participants’ genders, places of residence (northern Italy vs. central and southern Italy), or personal experiences with COVID-19 infection. Women scored higher than men on identification with family, *t*(844) = 2.53, *p* = 0.012, *d =* 0.19, and identification with friends, *t*(844) = 2.14, *p* = 0.033, *d* = 0.16. Women also reported more negative emotional reactions, *t*(844) = 6.10, *p* < 0.001, *d* = 0.47, higher compliance with protective behaviors, *t*(844) = 3.59, *p* < 0.001, *d* = 0.27, higher engagement in online actions, *t*(844) = 4.26, *p* < 0.001, *d* = 0.33, and higher support for people in need, *t*(844) = 3.47, *p* < 0.001, *d* = 0.27, than men. Finally, women scored higher than men on negative expectations about social life, *t*(844) = 2.03, *p* = 0.043, *d* = 0.16, and negative expectations about the pandemic impact on Italian and global economy, *t*(844) = 4.72, *p* < 0.001, *d* = 0.36. No other comparisons between women and men were significant (*t*s < 0.88, *p*s > 0.381).

Further analyses revealed a few variations due to place of residence, with respondents from center-southern regions scoring higher on humankind identification, *t*(844) = 5.51, *p* < 0.001, *d* = 0.39, and being more engaged in online actions, *t*(844) = 2.73, *p* = 0.006, *d* = 0.19, than respondents from northern regions. No other comparisons were significant (*t*s < 1.78, *p*s > 0.075). Finally, those who had personal experiences with COVID-19 reported higher support for people in need, *t*(844) = 2.92, *p* = 0.004, *d* = 0.23, and were less worried about the impact of the pandemic on their social lives, *t*(844) = −2.22, *p* = 0.027, *d* = −0.17, than respondents who had no personal experience with the virus. No other comparisons were significant (*t*s < 1.51, *p*s > 0.131).

### 5.2. Regression Analyses

Table 2 presents bivariate correlations among measures of social identification, negative emotional reactions, protective and prosocial behaviors, negative expectations for the future, political orientation, and age.

To examine the impact of social identification with different groups on the outcome variables, we conducted bootstrapped hierarchical regression analyses with 5000 resamples. For each outcome, we included in Model 1 age, gender (0 = man; 1 = woman), place of residence (0 = Northern Italy; 1= Central-Southern Italy), personal experience with COVID-19 (0 = no; 1 = yes), and political orientation. Model 2 added the social identification measures. Table 3 shows the results of these models.

#### 5.2.1. Negative Emotions

The hierarchical regression analyses revealed, for Model 1, significant effects of gender and political orientation on emotional reactions to the pandemic. Being female is associated with a more intensely negative emotional reaction. Moreover, the more right-wing oriented respondents were, the higher they scored on the negative emotional reactions measure. When the identification measures were entered in Model 2, identification with the family was associated with stronger negative emotional reactions, whereas European identification and humankind identification were related to weaker negative emotional reactions.

#### 5.2.2. Protective and Prosocial Behaviors

In Model 1, there were significant effects of gender on engagement in protective behaviors (with the exception of searching for information) and involvement in prosocial behaviors, with women being more likely than men to comply with the rules, engage in online actions, and support people in need. Age also turned out to be a significant predictor of protective and prosocial behaviors: As age increased, respondents were less compliant with the rules and showed increases in searches for information, engagement in online actions, and support for people in need. Living in center-southern regions was related to increased engagement in online actions. Finally, having personal experience with COVID-19 predicted higher support for people in need.

Model 2 revealed that, when the socio-demographic variables were controlled for, identification with family and identification with friends were both positively associated with compliance with the rules. Identification with family and national identification were positively related to searching for information, and national identification was positively related to engagement in online actions. Finally, humankind identification was positively associated with helping people in need.

#### 5.2.3. Negative Expectations Regarding the Future

In Model 1, being female was related to more intensely negative expectations regarding COVID-19′s impacts on one’s social life and the global economy. Older respondents reported more intensely negative expectations about the pandemic’s impact on their social lives but less intensely negative expectations about their economic situations. Living in center-southern regions was related to increased negative expectations about the economy. Moreover, the more right-wing oriented respondents were, the higher they scored on the negative expectations about their social life after COVID-19. When the identification measures were entered in Model 2, respondents who identified more with their family and their friends scored lower on the measure of negative expectations concerning their economic situations. Respondents who showed stronger European identification scored lower on negative expectations regarding their social lives.

### 5.3. Mediation Analyses

Given that emotions are strong predictors of behavioral reactions and the cognitive appraisals of a situation (e.g., [47]), we tested whether the index of negative emotional reactions related to the pandemic worked as a mediator of the relationships between social identifications and the outcome variables. We ran a series of mediation models with JASP [48], where the four identification measures were entered as predictors, the index of negative emotional reactions were entered as a mediator, and the socio-demographic variables (gender, age, place of residence, experience with COVID-19, and political orientation) were entered as covariates. The measures of protective and prosocial behaviors and the measures of expectations about the future were entered separately as outcome variables. The analyses revealed significant indirect effects through negative emotional reactions when the search for information was entered as outcome variable (*R*^2^ = 0.15). Specifically, there were significant indirect effects of identification with family, *b* = 0.03 (*SE* = 0.01), *p* = 0.021, 95% CI [0.01, 0.05], European identification, *b* = −0.02 (*SE* = 0.01), *p* = 0.007, 95% CI [−0.03, −0.01], and humankind identification, *b* = −0.03 (*SE* = 0.01), *p* = 0.007, 95% CI [−0.04, −0.01]. Similarly, there were significant indirect effects of identification with family *b* = 0.02 (*SE* = 0.01), *p* = 0.014, 95% CI [0.01, 0.04], European identification, *b* = −0.01 (*SE* = 0.01), *p* = 0.031, 95% CI [−0.02, −0.01], and humankind identification *b* = −0.01 (*SE* = 0.01), *p* = 0.041, 95% CI [−0.03, −0.01], on engagement in online actions (*R^2^* = 0.15). The analysis showed no indirect effects of negative emotional reactions on compliance with the rules (*R^2^* = 0.07) or helping people in need (*R^2^* = 0.07).

As regards negative expectations, our results revealed significant indirect effects of identification with family, *b* = 0.05 (*SE* = 0.02), *p* = 0.008, 95% CI [0.01, 0.09], European identification, *b* = −0.03 (*SE* = 0.01), *p* = 0.022, 95% CI [−0.06, −0.01], and humankind identification *b* = −0.03 (*SE* = 0.02), *p* = 0.031, 95% CI [−0.06, −0.01], on expectations about one’s social life (*R^2^* = 0.17). Similarly, there were indirect effects of identification with family, *b* = 0.03 (*SE* = 0.01), *p* = 0.012, 95% CI [0.01, 0.06], European identification, *b* = −0.02 (*SE* = 0.01), *p* = 0.028, 95% CI [−0.03, −0.01], and humankind identification *b* = −0.02 (*SE* = 0.01), *p* = 0.038, 95% CI [−0.04, −0.01] on expectations about one’s economic situation (*R^2^* = 0.09).

Thus, overall, emotional reactions played a mediational role in the relationships of family, European, and humankind identifications with some of the outcome variables. Family identification was related to stronger negative emotional reactions, which, in turn, accounted for the relationship between the predictor and increased search for information, online actions, and negative expectations regarding one’s social life and economic circumstances. European and humankind identification were instead related to lower levels of searches for information, online actions, and negative expectations for the future through reduced negative emotional reactions.

## 6. Study 2

Study 2 was conducted in Italy in June 2020, when limitations were partially loosened and people were trying to get back to their normal lives. At that time, the Italian government asked people to install and use *Immuni*, a contact-tracing App designed to help manage the second phase of the coronavirus emergency, whereas pharmaceutical companies were working globally to develop new vaccines and treatments. However, the fear of privacy violations represented a strong deterrent to the use of the tracing app [49]. Likewise, vaccine hesitancy for new products developed within a short period represented a severe challenge to achieving the coverage needed for population immunity [50].

The present study investigates how proximal and distant identifications predicted intentions to use the tracing app and to get vaccinated. Moreover, we appraise the role of negative emotions as mediators of the effects of social identifications on the willingness to install and use the tracing app and to get vaccinated.

## 7. Method

### 7.1. Participants

A total of 415 participants, recruited through social media and snowball sampling, volunteered for the study, which was presented as research on the psychological correlates of the pandemic. Ten participants did not provide consent to participate, and 49 were excluded for failure to complete the questionnaire. Six further participants were excluded, as they were not Italian, leaving a final sample of 350 participants (273 women; 77 men; *M*_age_ = 37.64 years; *SD* = 14.35 years; range = 18–72 years), all of Italian nationality. A sensitivity analysis conducted with G*Power showed that our sample was sufficient to detect small-size effects of *f^2^* = 0.11 (corresponding to *R*^2^ = 0.10), assuming an alpha of 0.05, and a power of 0.95.

### 7.2. Procedure

The project was approved by the Bioethical Committee of the University of Bologna. The data analyzed in this paper was collected between 3 and 18 June 2020. The questionnaire was administered via Qualtrics and included other measures (e.g., coping strategies, desires for stronger leadership that are not considered in the present paper), as they are unrelated to this study’s focus. The data from two measures included in this paper (national and European identifications) were also included in a manuscript submitted for publication and concerning a different issue [36]. Filling in the entire questionnaire required approximately 20 min. The completion was anonymous. Participants were first presented with measures of identification with a variety of social groups (i.e., family, friends, Italians, Europeans, and humanity) and then completed the measures of emotional reactions, intentions to use the tracing app, and intentions to be vaccinated against COVID-19. Participants then indicated whether they had contracted COVID-19 (*N* = 33; 9.4% of the sample) and whether a member of their family or a close friend had contracted the virus (*N* = 87; 24.9%). A total of 107 participants (30.1%) answered affirmatively to one or both questions (“personal experience with COVID-19”). Finally, each participant indicated their political orientation on an 11-point left–right scale, as in Study 1 (*M* = 3.71; *SD* = 2.05), and provided demographic information.

### 7.3. Measures

The measures of social identification and negative emotional reactions were the same as those of Study 1. Cronbach’s alphas were as follows: α = 0.94 for identification with the family; α = 0.90 for identification with friends; α = 0.81 for identification with Italians; α = 0.92 for identification with Europeans; α = 0.81 for identification with humankind; α = 0.77 for negative emotions. Intentions to use the tracing app and to be vaccinated against COVID-19 were measured through distinct items. Participants were asked the extent to which they intended to “use the tracing app (*Immuni*)” and “be vaccinated (when a vaccine will be available)” (1 = not at all; 5 = very much). 

## 8. Results

### 8.1. Preliminary Analyses

Table 4 shows the mean values of the main measures for the whole sample and individuated by gender, place of residence, and personal experience with COVID-19. Overall, participants showed greater identification with family than with all the other groups (*t*s > 3.28, *p*s < 0.001, Cohen’s *d*s > 0.18). Identification with friends was higher than national or European identification (*t*s > 16.23, *p*s < 0.001, *d*s > 0.87) but lower than humankind identification *t*(348) = −2.55, *p* = 0.011, *d* = −0.14. National identification was higher than European identification, *t*(348) = 4.00, *p* < 0.001, *d =* 0.21. Humankind identification was higher than national or European identification (*t*s > 18.82, *p* < 0.001, *d*s > 1.08).

A series of *t*-tests revealed that women reported more negative emotions than men, *t*(348) = 2.81, *p* = 0.005, *d* = 0.36. Participants from Center-Southern Italy reported higher humankind identification than those living in Northern Italy, *t*(348) = 2.53, *p* = 0.012, *d* = 0.30. Finally, participants who had personal experience with COVID-19 scored lower on national identification, *t*(348) = −2.08, *p* = 0.038, *d* = −0.30, than those who had no personal experience with the virus. There were no other differences due to respondent gender, place of residence, or personal experience with COVID-19 (*p*s > 0.107).

### 8.2. Main Analyses

Table 5 reports bivariate correlations among the measures of social identification, emotional reactions, intentions to use the tracing app and to be vaccinated, political orientation, and age.

As in Study 1, in order to examine the respective influences of the different social identifications on the outcome variables (emotional reactions, intentions to use the tracing app, and intentions to be vaccinated), we conducted a series of bootstrapped hierarchical regression analyses (5000 resamples), including age, gender, place of residence, personal experience with COVID-19, and political orientation in Model 1; we add the social identification measures in Model 2 (Table 6).

Regarding Model 1, women and younger respondents reported more intensely negative emotional reactions compared to men and older respondents. Moreover, as age increased, respondents less frequently reported intentions to be vaccinated. As for Model 2, as in Study 1, identification with one’s family predicted more intensely negative emotional reactions, which were instead negatively related to identification with friends. European identification turned out to be the only significant predictor of intentions to use the tracing app and to be vaccinated.

### 8.3. Mediation Analyses

As in Study 1, we ran a series of mediation analyses using JASP 0.16.2, where we entered social identification measures as predictors, emotional reactions as mediators, and intentions to use the tracing app and be vaccinated as separate outcome variables. The socio-demographic variables were entered as covariates. There were no indirect effects through emotions (*p*s > 0.117) with respect to intentions to use the tracing app (*R^2^* = 0.11). The analysis on intentions to be vaccinated (*R^2^* = 0.12) revealed indirect effects through emotions with respect to identification with family, *b* = 0.04 (SE = 0.02), *p* = 0.015, 95% CI [0.01, 0.07], and identification with friends, *b* = −0.05 (SE = 0.02), *p* = 0.009, 95% CI [−0.09, −0.01]. The analysis showed no other indirect effects.

## 9. Discussion

The present research aimed to verify the role of social identifications with proximal (family and friends) and distal groups (nation, Europe, and humankind) in helping individuals cope with COVID-19. To this aim, we conducted two studies in different stages of the pandemic evolution in Italy, focusing on emotional, psychological, and behavioral outcomes that were relevant at those times. Study 1, conducted at the beginning of the pandemic during the first strict lockdown, sought to identify patterns of identifications that protected individuals from negative emotions, favored compliance with protective behaviors, promoted prosocial behaviors, and mitigated negative expectations regarding the future.

Study 2 was conducted when containment measures were partially loosened, and the Italian government promoted new containment measures. Specifically, it aimed to assess the role of identifications in predicting negative emotions regarding the pandemic and participants’ intentions to use a tracing app and get vaccinated.

### 9.1. Reactions to the Pandemic and Engagement in Protective and Prosocial Behaviors

Results of Study 1 revealed that different social identifications had different relationships with emotional reactions to the pandemic. On the one hand, distal identifications were associated with reduced negative emotions: individuals’ feelings of belonging to the EU and humanity seem to buffer the negative impact of the COVID-19 outbreak and the lockdown at a psychological level. On the other hand, identification with a proximal group, such as family, was related to stronger negative emotions; that is, it appeared to exacerbate the negative feelings related to COVID-19 and its consequences.

Social identifications had varying relationships with compliance with protective and prosocial behaviors during the COVID-19 lockdown. Identifications with family or friends were predictors of compliance with the containment rules (washing one’s hands, maintaining social distancing, leaving home for essential reasons only). These findings align with results from Vignoles et al.’s study [4] conducted in the same period in the UK, in which identification with family predicted social distancing. It is likely that the threat of a virus for which there was not yet a cure or vaccine and which could infect significant others led to stricter respect for the containment measures. Moreover, as suggested by the literature, reference groups that are closely connected by proximity are more relevant and have a greater influence on normative behaviors than reference groups to which individuals are remotely connected [39].

A similar result emerged considering the search for information. The more individuals identified with family, the more they sought information on COVID-19 symptoms, read in-depth articles, and watched newscasts on the pandemic. Identification with Italians was also significantly related to this variable: The higher the degree of national identification, the more involved participants were in searching for information about the virus. The strength of national identification also predicted the frequency of online activities performed in support of public health containment measures.

These results can be explained by reference to certain contingent aspects of the situation our participants experienced. As mentioned, Italy was the first Western country facing a COVID-19 outbreak, and the eyes of the entire world were captured by images of the country’s empty cities. Every evening, many Italian citizens watched newscasts, where politicians and public health officials reported the numbers of infections and deaths while mobilizing the public to avoid certain behaviors that were no longer considered socially responsible and stressing the importance of acting together as members of a nation. In this phase, Italians exhibited a sharp increase in national identification and trust in their national government (e.g., [51]). The increased importance of one’s own country as a social identity for Italian citizens (e.g., [52]) may also explain why identification with Italians was related to searches for information and online actions supporting containment measures.

The findings concerning prosocial behaviors highlight the importance of identification with humanity. This distal identification implies that individuals fully expand the circle of people they care about, in contrast to other proximal identifications that clearly define the boundaries of the ingroup [53]. Specifically, this inclusive identification motivated prosocial behaviors toward all people in need and the elderly during the lockdown. This result confirms the consistent and powerful role of identification with all humanity in predicting cooperative and helpful behaviors toward other human beings during the pandemic [54], especially on behalf of vulnerable groups [55], even should they happen to live abroad [56].

As for expectations regarding the future, our results confirm the role of proximal and distal identifications in helping individuals deal with the pandemic’s negative consequences. European identification was related to lowered fears that one’s social life would never be the same after the pandemic, whereas proximal groups were probably perceived as meaningful sources of support that diminished concerns that the pandemic would have a negative economic impact on one’s life. Our analyses also revealed interesting gender differences. Women experienced higher levels of negative emotions and showed higher levels of concern about their social life and the economy compared to men. In alignment with the recent literature (e.g., [57]), women also showed higher compliance with containment rules and greater engagement in online actions and prosocial behaviors than men.

Additionally, participants’ age revealed unexpected but interesting patterns: Older respondents were more active in searching for information, online actions, and prosocial behaviors but were less compliant with containment rules. Moreover, older people were more worried about the consequences of the pandemic on their social life, presumably because they were more aware of their vulnerability as well as the risks of contracting the infection through social contact. However, the correlation between age and the negative economic consequences of the pandemic at the personal level revealed that older participants were less worried than younger participants about the possible effects of the pandemic on their family’s economic situation or work. This finding is likely due to the higher stability of older participants’ economic status.

Finally, the findings revealed that the relations between family, European, and humankind identification and some of the outcome variables were explained by negative emotions, even though these effects work in opposite directions. On the one hand, family identification increased the negative emotional reactions, which in turn increased the search for information, engagement in online actions, and negative expectations regarding the consequences of the pandemic on one’s social life and economic circumstances. On the other hand, European and humankind identification decreased search for information, engagement in online actions, and negative expectations about the pandemic’s consequences for one’s social life by reducing negative emotional reactions.

### 9.2. Intentions to Use the Tracing App and Get Vaccinated

Study 2 aimed to assess the role of identification with proximal and distal groups in predicting individuals’ negative emotions regarding the pandemic and their intentions to use a tracing app and get vaccinated.

In alignment with Study 1, the results showed that family identification was related to enhanced negative feelings about the COVID-19 outbreak. Taken together, the findings of the two studies are striking, given the literature showing the importance of family identification in improving mental health during the pandemic and helping individuals cope with stress (e.g., [38]). One possible explanation lies in the measure we adopted: We asked participants to indicate the degree of negative emotions (e.g., distress, fear) during the lockdown (Study 1), and although people were going back to “normal” life, there was no vaccine at that time (Study 2). Evans et al. [58] found that, for many parents, the social restriction and isolation measures placed unprecedented strains on parent–child relationships while simultaneously reducing possibilities for social support and respite. Additionally, parents reported concerns about the pandemic’s consequences for their children’s lives, including worries about their education, healthcare, sports, and social or other co-curricular activities. These effects are amplified in situations of financial instability or insecurity about employment [58]. Moreover, in extreme cases, the social isolation measures, the difficulties relating to leaving one’s home, the lack of contact with support networks, and the closing of social institutions increased instances of domestic violence [58,59] and substance abuse [60], thus making many familial environments significantly less safe.

Conversely, the other proximal identification, i.e., identification with friends, can buffer these negative emotions. Similarly, the mediation analysis conducted on the vaccination intention reveals that family identification increased the negative emotional reactions and, through it, the intention to get vaccinated. Identifying with friends, instead, decreased negative emotional reactions, and through it the propensities to get vaccinated. These findings align with recent research showing the stress-buffering role of friends’ social support during the pandemic (e.g., [61]) and confirm the importance of connecting with friends in times of imposed isolation to alleviate feelings of loneliness as well as emotional distress [62,63].

As in Study 1, women reported stronger negative emotions related to the pandemic. These results are in line with the literature according to which women show higher levels of post-traumatic stress disorder symptoms after pandemics, compared to men (e.g., [64,65]). However, another possible explanation could relate to men’s socialization processes. Men refrain from emotional expression because traditional masculine norms require them to resist showing vulnerable emotions. As Levant et al. [66] suggested, men who are discouraged from expressing their emotions show difficulty in recognizing, describing, and expressing emotions that reflect a sense of vulnerability (such as hurt, sadness, or fear) throughout their lives. Future research should investigate in-depth the origins of gender differences in the expression of emotions related to COVID-19.

The results highlight the importance of European identification in predicting intentions to engage in protective behaviors in the second phase of the pandemic: The more participants identified with Europe, the more they were willing to use the tracing app and get vaccinated. The associations between European identification and expectations about one’s social life after COVID-19 (Study 1) and the intentions to use the tracing app and to get vaccinated underline the importance of EU membership for those dealing with the pandemic. Not only does the EU have more means than individual member states in bargaining with drug companies for vaccine supplies and in providing financial support to the most hard-hit countries, but it also constitutes a further resource for individuals, as it can buffer their negative expectations about their social lives after COVID-19 and can promote responsible behaviors to fight the pandemic.

Remarkably, our analyses indicate that the likelihood of being vaccinated for COVID-19 decreases significantly with age. Consistent with Study 1, older participants seem less willing to conform to containment rules than younger participants. A possible explanation for vaccination hesitancy in older Italian people may lie in their mistrust of medicine and science [67]. Although Italy reached significant vaccination coverage [68], it is important to individuate those who are more hesitant and to shed light on the motivations behind that hesitancy.

### 9.3. Limitations and Future Directions

Our studies have some limitations. Firstly, we adopted the snowball sampling strategy, which is not based on a random selection of the sample. Thus, the study’s sample does not reflect the actual pattern of the general Italian population. In addition, we are aware that the online administration of the questionnaire may also have undermined the representativeness of the sample. Secondly, the cross-sectional data limits the possibility of solidly establishing cause-and-effect relationships between the analyzed variables. Thirdly, we conducted Study 2 at a time when the relevant vaccines were still unavailable, and the pharmaceutical companies were working globally to develop them. Now that the vaccination campaign has been implemented, scholars have found that vaccination propensities are negatively influenced by several factors, such as the spread of “fake news” and conspiracy theories, as well as a lack of trust in science (e.g., [50,67]). Thus, a future line of research should verify the role of multiple social identifications in limiting the negative influence of these factors in the context of the worldwide phenomenon of vaccination hesitancy [69].

### 9.4. Theoretical and Practical Implications

From a theoretical perspective, our studies contribute to the literature by showing that group memberships constitute “social cures” for individuals dealing with social crises, such as the COVID-19 pandemic [28]. Specifically, these two studies highlight that the positive effects of social identification are enhanced for individuals who belong to multiple groups [19,20]. In the first place, multiple identifications offer individuals continued protection in cases where one of them fails to respond positively to a stressful event. We found that identification with family was connected to increased distress during the lockdown and the second phase of the pandemic. At the same time, identification with friends and the European and humankind identifications act as buffers by opposing negative emotional reactions.

In the second place, the results show that proximal and distal identifications predicted different patterns of behaviors and expectations regarding the future. Proximal identities positively influence protective behaviors performed in the social contexts of everyday life (e.g., washing one’s hands, maintaining social distance). The more people identify with these groups, the more they perform behaviors that protect and avoid imperiling their significant others. In contrast, distal identifications predicted more general behaviors which play crucial roles in combating the pandemic: National identity promoted sensibilization online actions promoting containment measures; European identification predicted the propensity to use the tracing app and to get vaccinated; humankind identification predicted prosocial behaviors, such as material and social support for those in need.

The potential of distal identifications may have important practical implications for pandemic management. National leaders should act in ways that strengthen citizens’ national identity. At the communicative level, they may constructively use social identities in describing how to deal with critical events—for example, by employing national identity-based rhetoric rather than using individualistic appeals. Vignoles et al. [4] have compared these strategies by confronting the individual rhetoric of UK Prime Minister Boris Johnson with the national-community rhetoric of New Zealand Prime Minister Jacinda Ardern; they found that the latter, by attributing collective agency to the nation and by underling the group norms of mutual solidarity, was better able to mobilize the public to engage in protective actions than the former. Moreover, it is equally important that political leaders maintain and reinforce the idea that, despite the ideological differences among parties, they are all working to pursue a common goal (for similar reasoning, see [43]).

Our results also illuminate the importance of European identification. Previous research has shown that European identity constitutes a protective factor that increases trust in institutions and improves expectations regarding the future [35], while reducing the wishes for stronger leadership [36]. The present study adds to this evidence by showing the role of European identification in promoting behaviors that effectively combat the pandemic. Such a finding is even more important, considering that, at least in Italy, the EU is often seen as a rather distant entity, and its authorities are often depicted as “bureaucrats” (e.g., [70,71]). However, these findings point out that seeing the EU as a cohesive entity that is able to reach collective goals (for instance, when dealing with drug companies to secure vaccine supplies or when supporting member states in need) can reinforce individuals’ identification with it. This in turn has a series of positive repercussions for individuals’ abilities to cope with serious threats such as the pandemic. Thus, it is of fundamental importance that European leaders improve communications with EU citizens by showing that the EU is not a distant, overarching entity that enacts rules against its member states. At the same time, national leaders can promote citizens’ identification with the EU by nurturing the sense of being part of a superordinate common group when they communicate about EU decisions [44]. Finally, based on the present results, communicative strategies should strengthen individuals’ sense of belonging to the “human family”—which, despite the differences among national, ethnic, religious, and other criteria, constitutes an overarching group that might help us face common threats, such as the COVID-19 pandemic.

## 10. Conclusions

In conclusion, these studies highlight the crucial role of multiple group identifications in fostering well-being and promoting individuals’ resilient responses to COVID-19. Individuals who can rely on multiple meaningful identities will be better equipped to face the strains and uncertainties of pandemics and other global crises.

## Figures and Tables

**Table 1:** Descriptive statistics as a function of gender, place of residence, and experience with COVID-19 infection.

	Total	Gender		Place of Residence		Experience COVID-19	
		Women	Men	North	Center-South	Yes	No
Variables	M (SD)	M (SD)	M (SD)	M (SD)	M (SD)	M (SD)	M (SD)
1. Identification with family	6.32 (0.90)	6.37 (0.87)	6.20 (0.95)	6.28 (0.93)	6.39 (0.83)	6.32 (0.88)	6.32 (0.90)
2. Identification with friends	5.87 (1.00)	5.92 (0.96)	5.75 (1.10)	5.84 (1.02)	5.92 (0.97)	5.95 (0.96)	5.84 (1.02)
3. National identification	5.39 (1.05)	5.42 (1.04)	5.32 (1.06)	5.38 (1.02)	5.42 (1.09)	5.34 (1.07)	5.41 (1.04)
4. European identification	4.10 (1.55)	4.15 (1.52)	3.96 (1.63)	4.16 (1.53)	4.00 (1.58)	4.05 (1.58)	4.23 (1.46)
5. Humankind identification	5.83 (1.16)	5.85 (1.12)	5.78 (1.25)	5.66 (1.25)	6.11 (0.94)	5.79 (1.21)	5.85 (1.14)
6. Negative emotional reactions	2.97 (0.68)	3.05 (0.70)	2.74 (0.57)	2.97 (0.70)	2.97 (0.67)	2.96 (0.65)	2.97 (0.69)
7. Compliance with the rules	4.79 (0.52)	4.83 (0.46)	4.69 (0.64)	4.77 (0.57)	4.81 (0.41)	4.80 (0.56)	4.78 (0.50)
8. Search for information	3.46 (0.91)	3.47 (0.93)	3.41 (0.87)	3.42 (0.91)	3.53 (0.92)	3.47 (0.84)	3.45 (0.94)
9. Online actions	2.33 (1.11)	2.43 (1.14)	2.07 (0.98)	2.25 (1.10)	2.47 (1.12)	2.33 (1.12)	2.33 (1.11)
10. Helping people in need	3.03 (1.00)	3.11 (0.99)	2.84 (1.01)	3.02 (1.02)	3.05 (0.97)	3.20 (0.98)	2.97 (1.00)
11. Expectations about one’s social life	4.86 (1.30)	4.92 (1.31)	4.72 (1.27)	4.83 (1.25)	4.91 (1.37)	4.70 (1.30)	4.92 (1.30)
12. Expectations about one’s economic situation	4.44 (1.43)	4.46 (1.46)	4.39 (1.37)	4.42 (1.40)	4.48 (1.49)	4.46 (1.45)	4.44 (1.43)
13. Expectations about economy	6.41 (0.76)	6.48 (0.70)	6.21 (0.87)	6.38 (0.75)	6.45 (0.78)	6.44 (0.67)	6.39 (0.79)

Note: Measures from 1 to 5 and from 11 to 13 were on 7-point scale. Measures from 6 to 10 were on 5-point scale.

**Table 2:** Correlations among study variables.

Measures	2	3	4	5	6	7	8	9	10	11	12	13	14	15
1. Identification with family	0.27 ***	0.25 ***	0.08 *	0.19 ***	0.09 **	0.14 ***	0.13 ***	0.12 ***	0.07 *	−0.03	−0.13 **	0.04	0.01	0.13 ***
2. Identification with friends	1	0.25 ***	0.18 ***	0.22 ***	−0.01	0.17 ***	0.01	0.07 *	0.11 **	−0.05	−0.12 **	0.04	−0.01	−0.08 **
3. National identification		1	0.29 ***	0.24 ***	0.03	0.14 ***	0.17 ***	0.13 ***	0.10 **	−0.02	−0.08 *	0.05	0.04	0.03
4. European identification			1	0.21 ***	−0.10 **	0.09 **	0.01	−0.02	0.06	0.16 ***	−0.10 **	−0.01	−0.38 ***	−0.10 ***
5. Humankind identification				1	−0.08 *	0.08 **	0.08 *	0.12 ***	0.17 ***	0.04	−0.08 *	0.06	−0.26 ***	0.08 **
6. Negative emotional reactions					1	0.06	0.32 ***	0.20 ***	0.01	0.36 ***	0.21 ***	0.17 ***	0.11 **	−0.04
7. Compliance with the rules						1	0.07 *	0.08 *	0.04	.01	−0.09 *	0.07 *	0.01	−0.07 *
8. Search for information							1	0.24 ***	0.12 ***	0.18 ***	0.03	0.13 ***	0.05	0.12 ***
9. Online actions								1	0.25 ***	0.07 *	0.07	0.06	0.01	0.26 ***
10. Helping people in need									1	−0.03	0.04	0.01	0.01	0.11 **
11. Expectations about one’s social life										1	0.23 ***	0.31 ***	0.11 **	0.12 ***
12. Expectations about one’s economic situation											1	0.17 ***	0.06	−0.10 **
13. Expectations about economy												1	−0.03	−0.01
14. Political orientation													1	0.02
15. Age														1

Note: * *p* < 0.05; ** *p* < 0.01; *** *p* < 0.001.

**Table 3:** Hierarchical regression analysis testing the associations between social identification, emotional reactions, compliance with the containment rules, prosocial behaviors, and negative expectations.

	Negative Emotional Reactions	Compliance with the Rules	Search for Information	Online Actions	Helping People in Need	Expectations about One’s Social Life	Expectations about One’s Economic Situation	Expectations about Economy
	*β* [95%CI]	*β* [95%CI]	*β* [95%CI]	*β* [95%CI]	*β* [95%CI]	*β* [95%CI]	*β* [95%CI]	*β* [95%CI]
*Model 1*								
Gender	0.22 *** [0.24, 0.43]	0.13 *** [0.06, 0.25]	0.04 [−0.06, 0.21]	0.15 *** [0.23, 0.53]	0.11 *** [0.11, 0.40]	0.08 * [0.05, 0.44]	0.03 [−0.11, 0.32]	0.17 *** [0.16, 0.41]
Age	−0.05 [−0.01, 0.01]	−0.08 * [−0.02, −0.01]	0.12 *** [0.01, 0.02]	0.25 *** [0.01, 0.02]	0.11 *** [0.01, 0.02]	0.11 ** [0.01, 0.02]	−0.10 ** [−0.02, −0.01]	−0.02 [−0.01, 0.01]
Residence	0.04 [−0.05, 0.15]	0.07 [−0.01, 0.14]	0.03 [−0.02, 0.24]	0.09 * [0.05, 0.36]	0.04 [−0.06, 0.21]	0.02 [−0.14, 0.23]	0.04 [−0.41, 0.01]	0.07 * [0.01, 0.22]
COVID-19 (yes)	−0.02 [−0.13, 0.08]	0.01 [−0.08, 0.10]	0.04 [−0.07, 0.21]	0.03 [−0.10, 0.25]	0.11 ** [0.09, 0.40]	−0.07 [−0.41, 0.01]	0.01 [−0.09, 0.33]	0.03 [−0.07, 0.15]
Political orientation	0.12 *** [0.02, 0.06]	0.01 [−0.01, 0.02]	0.06 [−0.01, 0.05]	0.02 [−0.02, 0.04]	0.02 [−0.13, 0.15]	0.12 *** [0.03, 0.11]	0.07 [−0.01, 0.09]	−0.02 [−0.03, 0.02]
Model 1 *R^2^*	**0.06**	**0.02**	**0.02**	**0.10**	**0.04**	**0.04**	**0.02**	**0.03**
Model 1 *F*	10.83 ***	4.16 ***	3.80 **	17.78 ***	6.56 ***	6.41 ***	2.54 *	5.44 ***
*Model 2*								
Gender	0.22 *** [0.23, 0.42]	0.11 *** [0.04, 0.22]	0.03 [−0.08, 0.19]	0.14 *** [0.21, 0.50]	0.10 ** [0.08, 0.38]	0.09 ** [0.07, 0.46]	0.05 [−0.06, 0.37]	0.16 *** [0.15, 0.40]
Age	−0.07 [−0.01, 0.01]	−0.08 * [−0.02, −0.01]	0.10 ** [0.00, 0.01]	0.24 *** [0.01, 0.02]	0.11 *** [0.01, 0.02]	0.10 ** [0.01, 0.02]	−0.10 ** [−0.02, −0.01]	−0.03 [−0.01, 0.01]
Residence	0.04 [−0.04, 0.16]	0.05 [−0.02, 0.13]	0.04 [−0.05, 0.22]	0.03 **[0.02, 0.32]**	0.01 [−0.13, 0.15]	0.01 [−0.16, 0.23]	0.05 [−0.06, 0.37]	0.06 [−0.01, 0.21]
COVID-19	−0.01 [−0.12, 0.09]	0.01 [−0.09, 0.09]	0.04 [−0.06, 0.22]	0.07 * [−0.10, 0.25]	0.11 ** [0.08, 0.39]	−0.06 [−0.40, 0.02]	0.04 [−0.20, 0.26]	0.03 [−0.06, 0.15]
Political orientation	0.07 [−0.01, 0.05]	0.01 [−0.01, 0.02]	0.06 [−0.01, 0.05]	0.01 [−0.03, 0.04]	0.07 * [0.01, 0.07]	0.07 [−0.01, 0.09]	−0.04 [−0.03, 0.08]	−0.03 [−0.04, 0.02]
Identification with family	0.10 ** [0.02, 0.13]	0.09 * [0.01, 0.10]	0.09 * [0.02, 0.16]	0.03 [−0.03., 0.11]	−0.01 [−0.08, 0.07]	−0.04 [−0.16, 0.05]	−0.09 ** [−0.25, −0.04]	0.02 [−0.07, 0.11]
Identification with friends	−0.03 [−0.07, 0.03]	0.10 ** [0.01, 0.10]	−0.05 [−0.12, 0.02]	0.04 [−0.04, 0.12]	0.06 [−0.01, 0.13]	−0.02 [−0.13, 0.07]	−0.09 ** [−0.22, −0.02]	0.01 [−0.06, 0.06]
National identification	0.05 [−0.02, 0.09]	0.07 [−0.01, 0.08]	0.15 *** [0.06, 0.19]	0.10 ** [0.03, 0.17]	0.04 [−0.04, 0.11]	0.01 [−0.07, 0.11]	−0.02 [−0.12, 0.08]	0.05 [−0.03, 0.10]
European identification	−0.09 ** [−0.08, −0.01]	0.05 [−0.01, 0.04]	−0.01 [−0.06, 0.04]	−0.05 [−0.08, 0.02]	0.04 [−0.03, 0.07]	−0.12 ** [−0.17, −0.03]	−0.07 [−0.14, 0.02]	−0.05 [−0.07, 0.02]
Humankind identification	−0.08 ** [−0.09, −0.01]	0.02 [−0.01, 0.05]	0.04 [−0.03, 0.09]	0.06 [−0.02, 0.12]	0.16 *** [0.07, 0.20]	0.01 [−0.08, 0.09]	−0.02 [−0.12, 0.07]	0.03 [−0.04, 0.08]
Model 2 Δ*R^2^*	**0.02**	**0.04**	**0.04**	**0.02**	**0.03**	**0.01**	**0.03**	0.01
Model 2 Δ*F*	3.77 **	7.36 ***	6.27 ***	3.82 **	6.61 ***	2.52 *	5.19 ***	0.091

Note: Parameters are beta weights. Significant parameters are in bold. Gender was coded 0 = men, 1 = women. Place of residence was coded 0 = northern regions, 1 = central-southern regions. Experience with COVID-19 was coded (0 = no; 1 = yes). *** *p* < 0.001, ** *p* < 0.01, * *p* < 0.05.

**Table 4:** Descriptive statistics as a function of gender, place of residence, and experience with COVID-19 infection.

	Total	Gender		Place of Residence		Experience with COVID-19	
		Women	Men	North	Center-South	Yes	No
Variables	M (SD)	M (SD)	M (SD)	M (SD)	M (SD)	M (SD)	M (SD)
1. Identification with family	6.21 (1.17)	6.20 (1.26)	6.27 (0.79)	6.15 (1.25)	6.37 (0.97)	6.22 (1.09)	6.21 (1.21)
2. Identification with friends	5.80 (1.03)	5.81 (1.04)	5.75 (1.03)	5.77 (1.04)	5.87 (1.03)	5.76 (1.10)	5.82 (1.00)
3. National identification	4.73 (1.09)	4.75 (1.12)	4.65 (0.98)	4.70 (1.03)	4.81 (1.24)	4.55 (1.12)	4.81 (1.07)
4. European identification	4.43 (1.43)	4.44 (1.39)	4.40 (1.60)	4.41 (1.38)	4.47 (1.57)	4.38 (1.52)	4.45 (1.42)
5. Humankind identification	5.96 (1.03)	6.00 (1.06)	5.84 (0.94)	5.88 (1.07)	6.19 (0.89)	5.87 (1.10)	6.01 (1.00)
6. Negative emotional reactions	2.42 (0.63)	2.47 (0.66)	2.24 (0.51)	2.41 (0.64)	2.44 (0.62)	2.42 (0.62)	2.41 (0.64)
7. Using the tracing app	2.81 (1.43)	2.81 (1.45)	2.81 (1.37)	2.82 (1.42)	2.74 (1.45)	2.70 (1.49)	2.84 (1.40)
8. Getting vaccinated	3.896(1.31)	3.92 (1.29)	3.86(1.31)	3.94 (1.27)	3.69 (1.40)	3.89 (1.33)	3.86 (1.31)

Note: Measures from 1 to 5 were on 7-point scale; measures from 6 to 8 were on 5-point scale.

**Table 5:** Correlations among the study variables.

Measures	1.	2.	3.	4.	5.	6.	7.	8.	9.	10.
1. Identification with family	1	0.38 ***	0.30 ***	0.19 ***	0.18 ***	0.10	0.19 ***	0.07	−0.03	0.08
2. Identification with friends		1	0.33 ***	0.22 ***	0.31 ***	−0.14**	0.19 ***	0.09	−0.08	0.03
3. National identification			1	0.41 ***	0.33 ***	0.02	0.19 ***	0.06	−0.08	0.07
4. European identification				1	0.37 ***	0.02	0.23 ***	0.21 ***	−0.42 ***	0.02
5. Humankind identification					1	−0.06	0.13 *	0.07	−0.33 ***	0.04
6. Negative emotional reactions						1	0.08	0.20 ***	0.05	−0.18 **
7. Using the tracing app							1	0.40 ***	−0.13 *	0.03
8. Getting vaccinated								1	−0.09	−0.14 **
9. Political orientation									1	−0.01
10. Age										1

Note: * *p* < 0.05. ** *p* < 0.01. *** *p* < 0.001.

**Table 6:** Hierarchical regression analysis testing the associations between social identification, negative emotional reactions, and the intention to use the tracing app and to get vaccinated.

	Negative Emotional Reactions	Intention to Use the Tracing App	Intention to Get Vaccinated
	*β* [95%CI]	*β* [95%CI]	*β* [95%CI]
*Model 1*			
Gender	0.14 * [0.07, 0.36]	−0.01 [−0.39, 0.32]	0.06 [−0.12, 0.54]
Age	−0.17 ** [−0.02, −0.01]	0.03 [−0.01, 0.01]	−0.13 * [−0.02, −0.01]
Residence	0.07 [−0.05, 0.25]	−0.03 [−0.44, 0.26]	−0.07 [−0.52, 0.16]
COVID-19	0.042 [−0.11, 0.16]	−0.05 [−0.49, 0.19]	−0.01 [−0.32, 0.27]
Political orientation	0.06 [−0.01, 0.05]	−0.13 * [−0.17, −0.02]	−0.08 [−0.12, 0.01]
Model 1 *R^2^*	**0.05**	0.02	0.04 *
Model 1 *F*	3.92 **	1.46	2.48 *
*Model 2*			
Gender	0.15 ** [0.09, 0.37]	−0.01 [−0.39, 0.30]	0.07 [−0.09, 0.54]
Age	−0.17 *** [−0.02, −0.01]	0.02 [−0.01, 0.01]	−0.12 * [−0.02, −0.01]
Residence	0.07 [−0.04, 0.25]	−0.05 [−0.47, 0.19]	−0.07 [−0.12, 0.54]
COVID-19	0.02 [−0.11, 0.16]	−0.03 [−0.43, 0.23]	−0.01 [−0.53, 0.14]
Political orientation	0.06 [−0.02, 0.06]	−0.06 [−0.12, 0.04]	0.01 [−0.30, 0.30]
Identification with family	0.18 ** [0.05, 0.16]	0.11 [−0.01, 0.31]	0.05 [−0.07, 0.20]
Identification with friends	−0.21 *** [−0.21, −0.06]	0.10 [−0.02, 0.29]	0.04 [−0.10, 0.22]
National identification	0.04 [−0.05, 0.09]	0.06 [−0.09, 0.24]	−0.05 [−0.22, 0.11]
European identification	0.06 [−0.06, 0.08]	0.15 * [0.02, 0.27]	0.21 ** [0.08, 0.30]
Humankind identification	−0.04 [−0.12, 0.05]	0.01 [−0.18, 0.17]	−0.01 [−0.16, 0.14]
Model 2 Δ*R^2^*	**0.06**	**0.08**	**0.04**
Model 2 Δ*F*	4.02 ***	5.63 ***	2.81 *

Note: Parameters are beta weights. Significant parameters are in bold. Gender was coded (0 = men; 1 = women). Place of residence was coded (0 = northern regions; 1 = south-center regions). Experience with COVID-19 was coded (0 = No; 1 = Yes). *** *p* < 0.001, ** *p* < 0.01, * *p* < 0.05.

## Data Availability

The data presented in this study are available in the Open Science Framework at: https://osf.io/nxbsa/?view_only=b11224d3e6bf46f4976f8681a04cfeb9, accessed on 6 July 2022.
